# Hernia

**Published:** 1845-06

**Authors:** S. Gower

**Affiliations:** Surgeon


*Hernia.* By S. Gower, Esq., Surgeon.—When persons are thronging out of a theatre, one pressing another on, and each struggling to get first through a narrow opening which will permit but one at a time to pass, the theatre does not get emptied so soon as if all agreed to go out at such a pace as to leave the passage free for an uninterrupted sucession of one after another going through it quietly. If two stout persons, forced on by the crowd behind, should get wedged into that narrow passage, so that neither could stir, the obstruction, so long as it should remain unremoved, would be total, and prevent all egress to those behind. In the reduction of hernia by the taxis something not very dissimilar happens, from too violent pressure being made on the hernial tumor. I have seen violent efforts made at reduction, causing great pain to the patient; such efforts remitted, then repeated, to the injury of the contents of the sac, and then an operation talked of as the only method left. I have made gentle but firm pressure in the proper direction, just enough not to cause much pain. I have maintained such pressure, not relaxing it for a moment, but when one hand became wearied, substituting the other, till I have had the satisfaction of feeling the bowels slipping quietly, and almost imperceptibly, into their place, till the sac has been emptied. It may be set down as one rule of guidance, that you are not to make such pressure as will cause severe pain, for you thus not only cause pain, and do mischief to the parts, but crowd up and obstruct the narrow pass through which the bowels have to return. On making the requisite degree of pressure only, and patiently continuing it, a little air, or other contents of the intestine, will slip up, and a little bit of bowel with it, till you find the whole contents of the sac giving way under your hand. When this is percieved, the degree of pressure should be rather increased than diminished; but at any rate no relaxation of the hernia is felt to be on the move, till every fraction is returned. No good can be done by kneading a hernial tumor about like dough. *Lond. Lan.*

